# High molecular/low acetylated chitosans reduce adhesion of *Campylobacter jejuni* to host cells by blocking JlpA

**DOI:** 10.1007/s00253-024-13000-0

**Published:** 2024-01-24

**Authors:** Vanessa Kreling, Franco H. Falcone, Fabian Herrmann, Leon Kemper, Daniel Amiteye, Stefan Cord-Landwehr, Corinna Kehrenberg, Bruno M. Moerschbacher, Andreas Hensel

**Affiliations:** 1 Institute of Pharmaceutical Biology and Phytochemistry, University of Münster, Corrensstraße 48, 48149 Münster, Germany; 2 Institute of Parasitology, Justus Liebig University Giessen, Schubertstraße 81, 35392 Giessen, Germany; 3 Institute of Plant Biology and Biotechnology, University of Münster, Schlossplatz 8, 48143 Münster, Germany; 4 Institute of Veterinary Food Science, Justus Liebig University Giessen, Frankfurter Straße 92, 35392 Giessen, Germany

**Keywords:** Adhesion, Atomic force microscopy, *Campylobacter jejuni*, Chitosan, JlpA, Sacculus

## Abstract

**Abstract:**

Infections caused by *Campylobacter* spp. are a major cause of severe enteritis worldwide. Multifactorial prevention strategies are necessary to reduce the prevalence of *Campylobacter*. In particular, antiadhesive strategies with specific inhibitors of early host–pathogen interaction are promising approaches to reduce the bacterial load. An in vitro flow cytometric adhesion assay was established to study the influence of carbohydrates on the adhesion of *C*. *jejuni* to Caco-2 cells. Chitosans with a high degree of polymerization and low degree of acetylation were identified as potent antiadhesive compounds, exerting significant reduction of *C*. *jejuni* adhesion to Caco-2 cells at non-toxic concentrations. Antiadhesive and also anti-invasive effects were verified by confocal laser scanning microscopy. For target identification, *C. jejuni* adhesins FlpA and JlpA were expressed in *Escherichia coli* ArcticExpress, and the influence of chitosan on binding to fibronectin and HSP90α, respectively, was investigated. While no effects on FlpA binding were found, a strong inhibition of JlpA-HSP90α binding was observed. To simulate real-life conditions, chicken meat was inoculated with *C*. *jejuni*, treated with antiadhesive chitosan, and the bacterial load was quantified. A strong reduction of *C. jejuni* load was observed. Atomic force microscopy revealed morphological changes of *C. jejuni* after 2 h of chitosan treatment, indicating disturbance of the cell wall and sacculi formation by electrostatic interaction of positively charged chitosan with the negatively charged cell surface. In conclusion, our data indicate promising antiadhesive and anti-invasive potential of high molecular weight, strongly de-acetylated chitosans for reducing *C*. *jejuni* load in livestock and food production.

**Key points:**

*• Antiadhesive effects of chitosan with high DP/low DA against C. jejuni to host cells*

*• Specific targeting of JlpA/Hsp90α interaction by chitosan*

*• Meat treatment with chitosan reduces C. jejuni load*

**Supplementary Information:**

The online version contains supplementary material available at 10.1007/s00253-024-13000-0.

## Introduction

Bacteria from the genus *Campylobacter* are the most common zoonotic causes of severe bacterial gastroenteritis in humans worldwide, with an estimated 44 to 93 lethal cases per 10,000 humans (WHO 2012). *Campylobacter* species are predominantly occurring in the gastrointestinal tract of birds (frequently in chicken), warm-blooded mammals, or mollusks, typically without causing symptoms. In contrast, strong clinical symptoms are observed in case of infection of humans after oral intake of *Campylobacter*-contaminated products. During food processing, secondary contamination is often observed for food products and their primary packaging materials, but also contaminated drinking water is another important source of *Campylobacter* infections. *Campylobacter*-induced enteritis is characterized by strong diarrhoea, abdominal pain, cramps, fever, and fatigue. Long-term consequences of *C. jejuni* infection with poor therapeutic prognosis are autoimmune diseases such as Guillain-Barré syndrome, Miller-Fischer syndrome, or reactive arthritis (Kreling et al. 2020; Pope et al. 2007; Skirrow 1977; Ajene et al. 2013). *C. jejuni* is well equipped with manifold virulence factors to adapt to its different hosts: motility is mediated by highly specialized filaments, unusual *O*-glycosylation of the flagellum, as well as strongly pronounced chemotaxis and *quorum sensing*. Recognition of and adhesion to the host cells is mediated by the different outer membrane proteins, e.g. CadF (*Campylobacter* adhesion protein to fibronectin), FlpA (Fibrin-like-peptide A), and JlpA (*Jejuni* lipoprotein), interacting with complementary proteins of the host cells (e.g. fibronectin, heat shock protein 90α) and typically also by complex and variable *N*-glycosylation of surface proteins, which leads to the so-called phase variation (Kreling et al. 2020). Invasion into the host cell is mediated among others by *Campylobacter* invasion antigen (Cia) (Kemper and Hensel 2023). Destruction of host cells by cell cycle arrest is typically induced by cytolethal distending toxin (CDT) of *Campylobacter.* Prolonged intracellular survival is due to the formation of highly specialized *Campylobacter* containing vacuoles, which protects the bacterium from lysosomal degradation. For a recent review on *Campylobacter* pathogenicity factors and details of host–pathogen interaction, see Kemper and Hensel (2023) and Kreling et al. (2020).

Due to increasing antibiotic resistance of *Campylobacter*, alternative strategies are necessary to combat the infection. A recent study within a clinical setting indicated quinolone resistance of 59% and 4% of macrolide resistance (Baek et al. 2023). A recent review on factors associated with the occurrence of antibiotic resistant *Campylobacter* species in humans indicated travel, prior antimicrobial use, food consumption and handling, contaminated water consumption and exposure, and animal contact as important factors associated with the risk of infection with a resistant strain (Neustaedter et al. 2023). Especially meat production from livestock attributes to high resistance rates against a high number of different antibiotics (Nishino et al. 2023). As different species from the genus *Campylobacter* are able to exchange genetic material between each other and also with other bacteria genera, this facilitates the transfer of resistance and virulence (Bunduruș et al. 2023). Additionally, it has to be kept in mind that *Campylobacter* is widely hosted in different host species, which again can lead to exchange of genetic information by interconnecting these bacteria to different hosts, leading to negative consequences for the different hosts (Bunduruș et al. 2023).

Prevention strategies in livestock and food production are essential to reduce the infection risk, but also specific inhibition of the manifold virulence factors of *Campylobacte*r has been recognized as a promising tool. Especially, inhibitors of the bacterial adhesion to host cells, the so-called entry blockers or antiadhesive compounds, can help to reduce the early host–pathogen interaction (Sarshar et al. 2017; Schmuch et al. 2015; Selbach et al. 2023; Gottesmann et al. 2020a; Thöle et al. 2015; Deipenbrock et al. 2023). Thus, an antiadhesive strategy might also be interesting for reducing the *Campylobacter* load in livestock. Especially the treatment of animals during meat production with antiadhesive or anti-invasive compounds should reduce the *Campylobacter* load, which again would also reduce the excretion of pathogens by the faecal mass. This again could also be a benefit for reduced risk of contamination of water reservoirs, especially in the area of developing countries. This concept to interfere with the early host-*Campylobacter* interaction has been successfully proven by use of the scaffold protein azurin (Bakhshi et al. 2020), bovine oligosaccharides from milk (Douëllou et al. 2017), fucosylated human milk glycans (Douëllou et al. 2017), plant extracts from *Juniperus communis* (Klančnik et al. 2018), and polyphenol-enriched extracts from wine grapes (Klančnik et al. 2017). From the practical side of use in livestock, it has to be kept in mind that the use of highly complex oligosaccharides might be a specific approach, but probably not really realistic for practical use due to the high costs of isolation or synthesis of such compounds. In contrast, economically available carbohydrates should be developed, minimizing the problem of costs and availability. As the adhesion strategy of *C. jejuni* to host cells is strongly associated to carbohydrate-protein interactions, the use of antiadhesive glycans as potent entry inhibitors should be shifted into the focus for future drug development. For this, it has to be kept in mind that potential antiadhesive glycans should not get metabolized by enzymatic digest in the stomach or upper jejunum as they have to reach the lower intestinal compartments were *Campylobacter* is mainly located. Based on this, non-digestible polysaccharides are to be favoured. This aspect has to be kept in mind during compound development, as a recent report on glycans from *Abelmoschus esculentus* fruits has shown strong antiadhesive activity against *C. jejuni* under in vitro conditions, but animal experiments in chicken broilers did not show any positive effects concerning influencing the *Campylobacter* load, due to metabolic degradation of the test glycans by the birds (Lengsfeld et al. 2007). During a broad compound screening, mainly focused on carbohydrate-based natural products, strong antiadhesive properties of chitosans have been observed, which are well-known for their antimicrobial activity (Wagle et al. 2019; Ganan et al. 2009). Chitosans, which are mainly non-digestible polymers, are characterized by β-1,4-linked d-glucosamine residues, which in part can be *N*-acetylated. Chitosans, easily obtained from fully acetylated chitin, extracted, e.g. from crustaceans by partial deacetylation, comprise a complex group of polysaccharides differing in their degree of polymerization (DP) as well as their degree and pattern of acetylation (DA and PA). These structural parameters greatly influence the biological functionalities of chitosans so that detailed analyses of structure–function relationships are required to optimally exploit the biotechnological and pharmacological potential of these multi-functional biopolymers (Wattjes et al. 2020b; Cord-Landwehr et al. 2020).

Therefore, the following study aimed to investigate different chitosans with distinct structural features and showed that especially high DP/low DA chitosans exert strong antiadhesive activity against *C. jejuni*, which is due to the specific interaction with the outer membrane adhesin JlpA of the bacterium.

## Materials and methods

### Reagents and materials

Unless stated otherwise, solvents, reagents, and consumables were obtained from Merck (Darmstadt, Germany).

### Test compounds

Chitosans prepared by heterogeneous alkaline degradation (Muñoz et al. 2018) from α-chitin (shrimp shells or snow crab) or β-chitin (squid pen) with different molecular weights (MW), degree of polymerization (DP), dispersity (*Đ*), and degree of acetylation (DA) were used for functional testing and had been provided by Mahtani Chitosan Ltd., Gujarat, India, and HMC Heppe Medical Chitosan, Halle, Germany.

Details on origin and specifications of the different chitosans are given in Table 1. Test solutions were prepared from stock solutions of chitosans (10 mg/mL) in 100 mM sodium acetate/acetic acid buffer, pH 5.5.

**Table 1:** Overview of the selected chitosans and the respective analytical specifications. *MW* molecular weight [kDa], *DP* degree of polymerization, *DA* degree of acetylation [%], *Đ* dispersity. Analytical determination of specific properties according (Wattjes et al. 2020a; Schatz et al. 2003)

Chitosan	Batch	MW [kDa]	DP	DA [%]	*Đ*	Origin	Source
no designation	37H1327	495	2958	< 15	n.d	Sigma	Unknown
651	101,112	133	780	21.9	1.57	Mahtani	Shrimp shells
661	no batch designation	42*	250*	20	n.d	Mahtani	Shrimp shells
652	061212	78	480	8	2.19	Mahtani	Shrimp shells
70/20	212–100,715-03	95	556	23	1.74	HMC	Snow crab
80/20	212–201,015-04	106	629	17	1.36	HMC	Snow crab
90/20	212–16-1215–01	77	468	8	1.44	HMC	Snow crab
134	CF47	220	1360	≈ 1	1.72	Mahtani	Squid pen
114	20,170,125	496	3070	≈1	1.47	Mahtani	Squid pen

*661 is a mixture containing polymers and oligomers. The indicated value is only an approximated average value. *n.d.* not determined

### Bacteria and growth conditions

*Campylobacter jejuni* DSM 27585 (NCTC 11168, identification for quality control by PCR for *flpA* and *jlpA*) (Skirrow 1977; Parkhill et al. 2000) is routinely used for in vitro infection studies and exerts relevant virulence-associated pathogenicity factors (Gaynor et al. 2004). *C. jejuni* was cultured under microaerophilic conditions at 5% O_2_, 10% CO_2_, and 85% N_2_ using CampyGen bags (Thermo Fisher, Wesel, Germany) in an anaerobic chamber. The culture was carried out in a biphasic medium on Mueller–Hinton blood agar (MHB), supplemented with 2 mL of liquid medium brain heart infusion (BHI) for 48 h at 37 °C (Field et al. 1993). Correlation of OD to CFU was performed by plate counting of 10 × dilution series within 3 independent biological experiments. OD_600_ 0.2 is correlated to 5 × 10^9^ CFU/mL.

*C. jejuni* isolates LH90 and LH187 were obtained from Institute of Veterinary Food Science, University of Giessen (Germany).

## Fluorescence-labelling of *C. jejuni*

*C. jejuni* was labelled using carboxyfluoresceindiacetat-5/6-succinimidylester (CFDA-SE) (Bensch et al. 2011). Fifty microlitres of the CFDA-SE solution (5 μM) was added to 450 µL of the bacterial suspension (OD_600_ 0.4 in PBS), and the suspension was incubated for 30 min at 37 °C under shaking (350 rpm). Bacteria were centrifuged (4,725 × g, 5 min), and the resulting pellet was washed 2 × with medium. Between each washing step, the medium was left for 10 min at 37 °C in the bacterial suspension in order to maximize excess CFDA-SE reaction with components of the medium.

### Cell culture

Human, adherent Caco-2 epithelial cells (ATCC HTB-3), isolated from colon tissue from a 72-year old, white, male with colorectal adenocarcinoma (Jumarie and Malo 1991), were cultured in Dulbecco’s Modified Eagle’s Medium DMEM, 4.5 g/L glucose, with GlutaMAX Supplement (Thermo Fisher, Germany), without sodium pyruvate and 3.7 g/L NaHCO_3_ (PAN Biotech, Aidenbach, Germany), supplemented with 10% (v/v) foetal calf serum (FCS) (Merck, Darmstadt, Germany) and penicillin/streptomycin 0.5% (Biochrom, Berlin, Germany), at 37 °C/10% CO_2_. For experiments, passages 32 to 75 were used.

### Cell viability (Mosmann 1983)

For cell viability testing (MTT assay) 10,000 Caco-2 cells per well were seeded into a 96-well plate and incubated for 48 h at 37 °C/10% CO_2_. The medium was removed, and cells were washed with PBS. Two hundred microlitres of test solutions were added, and the culture was incubated for 48 h. The cells were washed 3 × with PBS. 50 µl of 3-(4,5-dimethylthiazol-2-yl)-2,5-diphenyltetrazolium bromide solution in PBS (5 mg/mL) were added, followed by 4-h incubation. The solution was removed, and formazan crystals were dissolved in 50 µL DMSO under exclusion of light. Absorbance was measured at λ = 595 nm against λ = 690 nm.

### Proliferation assay

By measuring the OD and subsequent CFU counting of a 10 × dilution, the OD_600_ of 0.2 was correlated to approximately 5 × 10^9^ CFU/mL (Khanna et al. 2006).

For the determination of the influence of test compounds on the growth of *C. jejuni* bacteria from overnight, the culture was suspended in 5 mL medium. The suspension was adjusted to an OD_600_ of 0.04. Sample dilutions were prepared in BHI medium at 2 × the target concentration. One hundred microlitres of the bacterial suspension was added to 96-well plates. Norfloxacin (100 μg/mL) served as positive control and medium only was used as growth control. Incubation was performed for 48 h under microaerobic conditions. The OD was determined at *λ* = 600 nm.

## Quantitative in vitro adhesion assay of *C. jejuni* interaction with Caco-2 cells by flow cytometry

*C. jejuni* culture in PBS was adjusted to an OD_600_ of 0.4. The bacteria were fluorescence-labelled with 5 µM carboxyfluorescein diacetate-5/6-succinimidyl ester (CFDA-SE) (Bensch et al. 2011). For preincubation, the labelled *C. jejuni* were incubated together with the test compounds in antibiotic-free medium at different concentrations for 2 h under microaerobic conditions at 37 °C. Bacteria were washed 3 × with PBS. Caco-2 cells were incubated in 6- or 12-well plates until 80 to 90% confluence was reached, washed with PBS, and overlaid with antibiotic-free cell medium. Adhesion of the bacteria to the host cells was achieved within a 1-h incubation at 37 °C/10% CO_2_. Subsequently, non-adherent bacteria were removed from the eukaryotic cells by washing 3 × with PBS. Cells with adherent bacteria were detached from the plate by addition of trypsin/EDTA and incubation for 3 min at 37 °C/10% CO_2_. The reaction was stopped by addition of antibiotic-free cell medium, and the suspension was centrifuged (500 × g, 5 min). The resulting pellet was resuspended in 500 µL DMEM and transferred to tubes for flow cytometric determination using *λ*_ex_ = 525, *λ*_*em*_ = 540 (CytoFlex, Beckmann Coulter, Krefeld, Germany). The adhesion of *C. jejuni* to host cells was quantified by using the median of the resulting fluorescence intensity. Prior to compound testing, investigation of potential quenching effects was performed.

### FlpA and JlpA protein expression

Chromosomal DNA from *C. jejuni* DSM 27585 was extracted by use of DNAeasy kit (Qiagen, Hilden, Germany) according to the instructions of the manufacturer. Genes coding for the *Campylobacter* adhesins *jlpA* and *flpA* were amplified by PCR using JumpStart Taq DNA Polymerase (Merck, Darmstadt, Germany), denaturing at 94 °C/10 s, annealing 64–45 °C, 30 s, 35 cycles, elongation 72 °C, 1 min, final elongation 72 °C, 5 min. Primers added 5′ and 3′ restriction sites for Nde I/XbaI (*flpaA*) and NdeI/Pst I (*jlpA*), respectively. Amplicons were restriction digested with the above endonucleases, purified on 1% (w/v) agarose gel in 0.5 × TBE, isolated by use of Monarch DNA Gel Extraction Kit (NEB, Frankfurt, Germany) and cloned into pCOLD I (Agilent Technologies, Rheine, Germany). Sequence identity of the so obtained recombinant plasmids was ensured by sequencing (Microsynth Seqlab, Göttingen, Germany). Details for primer and plasmids are displayed in Supplementary Table S1. The recombinant pCOLD plasmids were transformed into *E. coli* ArcticExpress ultracompetent cells (Agilent Technologies, Rheine, Germany) according to the instructions of the manufacturer, which subsequently expressed the target proteins in high yield at low temperatures. Bacteria were subcultured in 800 mL LB medium (without antibiotics) for 3 h at 30 °C until an OD_600_ of 1.2 was reached. The cultures were cooled down to 11.5 °C for 10 min, and protein expression was induced by addition of isopropyl-β-D-thiogalactopyranoside (IPTG, 1 mM). Incubation of the bacteria for protein expression was performed for 48 h at 11.5 °C under shaking at 250 rpm. Bacteria were centrifuged (4000 × g, 30 min, 4 °C), and the pellet was resuspended in a 1:10 ratio in lysis buffer I (PBS, containing lysozyme 1 mg/mL and 1 tablet of Roche cOmplete protease inhibitor cocktail per 50 mL). The bacterial pellet was lysed at 37 °C for 30 min, frozen 3 × with liquid nitrogen, rapidly thawed and sonicated on ice by use of an ultrasonic probe (5 × , 50% power, 30 s ON, 60 s OFF). After centrifugation (3,200 × g, 30 min, 4 °C), proteins were analysed by SDS-PAGE and western blotting. Purification was performed by immobilized metal ion chromatography on HisTrap HP stationary phase (7 × 25 mm, volume 1 mL) using ÄKTAstart FPLC system (GE Healthcare, Freiburg, Germany) and elution with imidazole gradient (flow 1 mL/min), fraction size 1 mL. Desalting of fractions was performed using an Amicon Ultracel-10 unit (Merck, Darmstadt, Germany). Protein sequences were determined after tryptic digest by HPLC–MS.

### Sandwich ELISA

An in-house solid-phase sandwich ELISA monitoring the influence of chitosans on the interaction of recombinant FlpA to fibronectin (FN) and JlpA to Hsp90α was developed (Gottesmann et al. 2020b; Konkel et al. 2010). Maxisorb plates (Thermo Fisher, Wesel, Germany) were coated with plasma FN (1 µg, PromoCell, Heidelberg, Germany) or 100 ng heat shock protein HSP90α (EnzoLife Science, New York, USA) overnight. After blocking with 3% (w/v) BSA solution in PBS-T for 1 h, the recombinant proteins FlpA (7.5 µg) or JlpA (0.5 µg) were incubated with the test compounds in a dilution series for 90 min within a coincubation protocol. All volumes given in the following protocol are related to 100 µL/well.

Protein bound to its respective ligand was quantified by subsequent incubation with 100 µL/well of a primary monoclonal 6 × His-tag antibody (Invitrogen, Rockford, Canada, 1:2,000 dilution, 2 h incubation at room temperature) and 100 µL/well of a secondary polyclonal goat-anti-mouse IgG (H + L) horseradish peroxidase (HRP) conjugate antibody (Invitrogen, Rockford, Canada, 1:5,000 dilution, 1 h incubation at room temperature).

After the addition of 50 µL of single-component TMB peroxidase EIA substrate (BioRad, Munich, Germany), HRP converts the substrate to a blue dye. The reaction was stopped by addition of sulphuric acid (50 µL, 2.5 M), and detection was made at *λ* = 450 nm. Between each incubation step, the samples were washed 3 × with 100 µL PBS-T wash buffer, to remove unbound proteins and antibodies. All samples were tested in at least 3 independent experiments. Data are expressed as percentage of relative absorbance, with 0% representing the blank value for a well and 100% representing the maximum value in a well.

### Invasion assay by confocal microscopy

Using a triple staining protocol, the interaction of bacteria and host cells can be distinguished and differentiated. Bacteria were labelled green with CFDA-SE, host cell nuclei were labelled blue using 4,6-diamidin-2-phenylindol fluorescent DNA stain (DAPI), and the host cell membrane was labelled red using wheat germ agglutinin (WGA)-Alexa Fluor 594 conjugate (Frutos-Grilo et al. 2023). With this double staining of the host cells, a distinction between the inner and outer levels is possible. Thus, the determination of both adherent and invaded bacteria in one system is possible.

For visualization of the adhesion and invasion of CFDA-SE-labelled *C. jejuni* by confocal laser scanning microscopy, a glass coverslip (⌀ 13 mm, VWR) inside a 12-well plate was treated with 200 µL of a solution of collagen I from rat tail (ChemCruz) (4 mg/mL in 0.02 M acetic acid) for 1 h under UV light at *λ* = 254 nm). Subsequently, the coverslips were air-dried. Afterwards, 1 × 10^5^ Caco-2 cells/well were seeded into the 12-well plates with the coverslips and incubated at 37 °C/5% CO_2_ for 48 h until 80 to 90% confluence. Cells were washed 1 × with PBS. After incubation with 1 mL DAPI (Carl Roth (0.35 Mol/L in PBS, staining with 200 μL) for 10 min and AlexaFluor 594 conjugated to wheat germ agglutinin (WGA, Invitrogen) (25 μg/200 µL in PBS, staining with 50 μL for 15 min) at 37 °C/5–10% CO_2_ under light protection, cells were washed 3 × with PBS and incubated with 1 mL of DMEM/FCS and CFDA-SE labelled *C*. *jejuni* at a BCR of 100:1.

For monitoring bacterial adhesion 1 h of incubation, for invasion assay, a 5-h period of incubation was performed. Subsequently, supernatants were removed, and the cells were washed 3 × with PBS, fixated for 15 min by use of paraformaldehyde 4%, and washed 1 × with PBS and 1 × with water and mounted onto slides using Fluoromount-G (Invitrogen). Samples were visualized under the confocal microscope LSM800 Axio Observer Z1 (Carl Zeiss) using AF568 (*λ*_Detection_ 575–700 nm), FITC (*λ*_Detection_ 505–575 nm), and DAPI (*λ*_Detection_ 400–505 nm) channels and z-stacking of 15 slices per image. Micrographs were treated with ImageJ software and analysed by the Cross Section Viewer plugin.

## Influence of chitosan 134 on *C. jejuni* load on chicken meat

The qualitative detection of *Campylobacter* in meat samples was carried out according to (Butcher and Stintzi 2017) and the German Governmental Methods for the detection of *Campylobacter* in food (DIN Normenausschuss Lebensmittel 057–01-06 AA 2017). Three independent series of experiments were carried out with different fresh chicken meat sample, obtained from different sources (slaughterhouse, farm shop).

Meat samples of 1 g were used. Four sample groups were created: one group without any treatment for determination of the endogenous *Campylobacter* load [designation: (− / −)], one group was treated with chitosan 134 [designation: (− / +)], one group was inoculated with a defined load of *C. jejuni* DSM 27585 [designation: (+ / −)], and another group was inoculated with *C. jejuni* and treated with chitosan 134 [designation: (+ / +)].

Inoculation with *C. jejuni* DSM 27585 was performed by use of a bacterial suspension (OD_600_ = 0.2, equivalent to approximately 1.25 × 10^8^ CFU) from which 1 mL/g meat was used, followed by a 1 h incubation period. Subsequently, the sample group [+ / +] was incubated with chitosan 134 (2 mL of a 10 mg/mL solution per gram of meat sample) for 2 h or buffer in case of [+ / −] group. Thus, the following four groups were examined both qualitatively and quantitatively for the presence of *C. jejuni* (“negative” means: without addition or without inoculation; “positive” means: with addition of with incubation):

- *C. jejuni* DSM 27585 negative, chitosan 134 negative (−/−)
- *C. jejuni* DSM 27585 negative, chitosan 134 positive (−/+)
- *C. jejuni* DSM 27585 positive, chitosan 134 negative (+/−)
- *C. jejuni* DSM 27585 positive, chitosan 134 positive (+/+)

Samples were washed 2 × with 1 mL PBS between each incubation step and then cut into approximately 0.5 cm^3^ pieces. From these pieces, the initial dilution was prepared. The sample was incubated with 9 × the amount of Preston Bouillon without antibiotic additives at 42 °C in a microaerophilic atmosphere (initial dilution). After 5 h, 700 µL of the antibiotic/antimycotic solution (polymyxin-B-sulfate 5000 I.U., rifampicin 10 μg, trimethoprim lactate 10 μg, amphotericin B 10 μg, EtOH 5 mL) were added. Incubation was continued for 20 h. The suspension was centrifuged (450 × g, 5 min). For isolation of *Campylobacter*, 100 µL of the solution was plated on modified charcoal cefoperazone deoxycholate (mCCD) agar. Plates were incubated at 42 °C for 48 h in a microaerobic atmosphere and examined for the presence of *Campylobacter* colonies. At least one *Campylobacter* colony was streaked out for subculturing on antibiotic-free Columbia Blood Agar. The so obtained culture was incubated for 48 h in a microaerobic atmosphere. The presence of *C. jejuni* was monitored by multiplex PCR.

Quantitative analysis of *Campylobacter* was performed according to (DIN Normenausschuss Lebensmittel 057–01-06 AA 2017). Three decimal dilutions were prepared from the initial dilution using Preston broth. Subsequently, mCCD plates were inoculated with 100 µL of the test solutions and incubated under microaerophilic conditions at 42 °C for 48 h. After incubation, plates were examined for the presence of typical *Campylobacter* colonies, which were counted, compared between groups, and identity confirmed by PCR.

## Multiplex-PCR for identity testing of *C. jejuni* (Butcher and Stintzi 2017)

For identification of *C. jejuni*, multiplex PCR was performed using the following genes: Hippuricase (*hipO*) with 344 bp is characteristic of *C. jejuni*, aspartokinase (*asp*) with 500 bp is characteristic of *C. coli*, and a gene for 16S rRNA with 1062 bp is non-specific for prokaryotes. The primers used are listed in Supplementary Table S1. Putative colonies were suspended in 20 µL of lysis buffer in a 1.5-mL tube and incubated at 100 °C for 15 min. The lysate was cooled on ice for 5 min, and 180 µL of nuclease-free water were added. After centrifugation (14,000 g, 5 min, 4 °C), the lysates were stored at − 20 °C. The amount of DNA in the lysates was determined photometrically. For PCR, 1–2 µl of the lysate were used, depending on the amount of DNA.

## Atomic force microscopy of *C. jejuni* cells under ambient conditions in air

In order to prepare *C. jejuni* cells for AFM imaging, fixation of bacterial suspension cultures was carried out by the application of fresh paraformaldehyde solution (4% in PBS, pH 7.2) for 15 min under gentle agitation. The samples were subsequently washed once with PBS and twice with Aqua Millipore. Finally, 10 µL of suspension were transferred on glass slides and dried under ambient conditions. The dried samples were afterwards washed thrice with Aqua Millipore and finally dried under constant air flow for subsequent AFM investigation. AFM imaging was carried out in intermittent contact mode under ambient conditions in air using a Bruker Dimension 3100 AFM (Bruker, Karlsruhe, Germany), equipped with soft tapping cantilevers (HQ:NSC14 Al BS, µmesh, Sofia, Bulgaria).

## Isolation and purification of *C. jejuni* peptidoglycan sacculi

Isolation and purification of *C. jejuni* peptidoglycan sacculi was carried out from bacterial suspension cultures by resuspending and boiling PBS-washed bacterial pellets in 3 mL of 8% (v/v) SDS solution at 95 °C and 350 rpm for 6 h. The samples were subsequently centrifuged (10.000 rpm for 10 min) at room temperature and repeatedly washed with PBS until no bubbles of remaining SDS were recognizable. After an additional centrifugation, the supernatant was discarded, and the pellet resuspended in 500 µL of 100 mM TRIS–HCl buffer, pH 7.4. Remaining sample impurities were then removed by the application of α-amylase (100 µg/mL at 37 °C for 60 min) and pronase-E (100 µg/mL at 60 °C for 90 min). After enzymatic digestion, samples were resuspended and boiled in 3 mL of 1% (v/v) SDS solution, removing the added enzymes as well as the solubilized sugar and protein components. The SDS was finally removed until no SDS was detected, and the resulting peptidoglycan pellet was resuspended in Aqua Millipore and stored at 4 °C for subsequent AFM investigations.

## Atomic force microscopy of isolated *C. jejuni* peptidoglycan sacculi

Prior to atomic force microscopy imaging, the isolated *C. jejuni* sacculi were immobilized on MICA discs. To do so, a V1 grade MICA disc (Nanoandmore, Wetzlar, Germany) was freshly cleaved, and a drop of 100 µL poly-L-lysine solution was added (0.1 mg/mL, poly-L-lysine supplied by Merck KGaA, Darmstadt, Germany). After 30 min, the drop was removed by gentle washing with Aqua Millipore for altogether three times. The MICA discs were afterwards dried under constant air flow. Ten microlitres of the previously described sacculus suspensions were afterwards added and allowed to settle for 30 min. The samples were subsequently washed thrice with Aqua Millipore, dried under constant airflow, and finally imaged by intermittent contact mode AFM under ambient conditions in air using a Bruker Dimension 3100 AFM (Bruker, Karlsruhe, Germany), equipped with soft tapping cantilevers (HQ:NSC14 Al BS, µmesh, Sofia, Bulgaria).

### Statistical analysis

Results are expressed as mean value (MV) ± standard deviation (SD). Mean values were compared by a one-way ANOVA test followed by a Tukey’s test for multiple comparisons. A *p*-value < 0.05 compared to the negative control was considered statistically significant. IC_50_ values were calculated with GraphPad Prism® Vers. 7 (GraphPad Software, Inc., La Jolla, USA).

## Results

### Chitosan exerts antiadhesive activity against *C. jejuni*

Within a screening of carbohydrate-based natural products on the interaction of *C. jejuni* with Caco-2 cells strong antiadhesive effects were detected for a standard chitosan, obtained from a commercial supplier. Subsequently, detailed investigations were performed for a deeper understanding of potential structural features of these polymers, which are necessary to exert antiadhesive effects against *C. jejuni*.

For this, a library of chitosans was established with polymers differing in molecular weight (MW), degree of polymerization (DP), dispersity (*Đ*), and degree of acetylation (DA) (Table 1). To define optimized chitosan concentrations for the adhesion assays, which do not harm the bacteria and the host cells, systematic preinvestigations were performed.

The influence of the chitosans (1.25 to 5 mg/mL) on cellular viability of Caco-2 was investigated after a 48-h incubation by MTT assay (Mosmann 1983) (Table 2). All chitosans tested reduced the cellular viability at the 5 mg/mL concentration level. Interestingly, chitosans 134 and 114, which are characterized by high DP and low DA (< 2%), did not affect the viability of the host cells at lower concentration. The same was due for polymers 651, 652, and 661, representing polymers with medium DP and relatively high DA (10 to 20%). In contrast, slightly reduced viability was observed at 2.5 and 1.25 mg/mL for chitosans with reduced molecular weight. From these data, it is concluded that viability of Caco-2 cells is mainly influenced by chitosans with lower DP and higher DA.

**Table 2:** Influence of different chitosans on the relative cell viability [%] of Caco-2 cells (MTT assay). Cells were incubated for 48 h with the test substances. Relative data are related to the untreated control (= 100%). Values represent the mean ± SD from *n* = 3 independent experiments with 4 technical replicates

Chitosan	Relative viability [%]		
	5 mg/mL	2.5 mg/mL	1.25 mg/mL
134	29 ± 12	88 ± 47	98 ± 45
114	63 ± 58	113 ± 45	134 ± 68
90/20	26 ± 13	50 ± 21	63 ± 14
80/20	26 ± 12	116 ± 35	102 ± 62
70/20	43 ± 36	80 ± 8	65 ± 24
651	22 ± 16	106 ± 23	81 ± 6
652	45 ± 40	115 ± 24	72 ± 21
661	32 ± 18	57 ± 29	96 ± 12

Subsequently, the influence of the different polymers on the proliferation of *C. jejuni* DSM 27585 was investigated. Bacteria were incubated for 48 h with chitosans, followed by removal of the test compounds and further 48 h of incubation in unsupplemented media. The relative proliferation data of the described experiments are displayed in the Supplementary Table S2. All polymers tested exert antiproliferative effects at concentrations of 5 and 2.5 mg/mL against *C. jejuni*. This influence is independent on the respective DA and DP. For all test compounds bacteriostatic effects are obvious, bactericidal effects were not observed.

During these investigations, the high standard deviation was disturbing, which could not be reduced by increasing the number of replicates. This high SD was not observed during testing of other polysaccharides (glucans, mannans, pectins). Microscopic investigations of chitosan-treated bacteria showed a quite high degree of agglutination towards cluster formation, which explains the high SD values.

For the subsequent investigations, potential antiadhesive effects of chitosans against *C. jejuni* concentrations of ≤ 1 mg/mL were selected so that cytotoxic effects against host cells and bacteria can be excluded. However, it should be noted that the studies described above on the influence of the test substances on bacterial proliferation or on the viability of the host cells were carried out over 48 h, which means over a quite long contact period. In contrast, investigations on host–pathogen interaction involve only a short contact time (60 to 120 min) with the test compounds. For this reason, it is assumed that chitosan concentrations of ≤ 1 mg/mL will not lead to relevant changes in host cells and bacteria.

For investigation of potential antiadhesive effects of the different chitosans, CFDA-SE labelled *C. jejuni* were preincubated with the test compounds (10, 100 μg/mL) for 2 h. Subsequently, Caco-2 cells were incubated for 1 h with the pretreated and labelled bacteria (bacteria cell ratio, BCR 100:1). Non-adherent bacteria were removed and after trypsinization the relative bacterial adhesion was quantified by flow cytometry.

For evaluation, the different chitosans were compared group wise for potential antiadhesive effects in relation to the respective DA and DP (Table 3). Chitosans 651 and 652 with comparable DP, but different DA (20% resp. 10%) exerted only low antiadhesive potential. This hypothesis is confirmed by the antiadhesive potential of chitosans 90/20 *vs.* 80/20: similar DP, but different DA (8 *vs.* 19) indicated significant activity at 10 and 100 μg/mL for the low acetylated polymer Table 4.

**Table 3:** Relative adhesion of *C. jejuni* (DSM 27585) to Caco-2 cells, related to the untreated control (= 100%) after 2-h preincubation of the bacteria with chitosans with different DP and DA (BCR 100:1). Data represent mean ± SD from *n* = 3 independent experiments with *n* = 2 technical replicates each

Chitosan	651	652	661	90/20	80/20	70/20	114	134
MW [kDa]	145	120	19	79	98	64	463	289
DA [%]	20	10	20	8	19	23	1	1
Concentration [µg/mL]	Rel. Adhesion ± SD [%]							
100	74 ± 40	72 ± 13 *p* = 0.0227	96 ± 8	48 ± 5 *p* < 0.0001	70 ± 13 *p* < 0.0154	97 ± 16	71 ± 40	36 ± 23 *p* < 0.0078
10	91 ± 6	81 ± 18	104 ± 12	84 ± 4 *p* < 0.0038	84 ± 17	108 ± 3	90 ± 7	92 ± 42

**Table 4:** Relative adhesion [%] related to the untreated control (= 100%) of *C. jejuni* (DSM 27585, isolates LH 90 and LH 187) to Caco-2 cells (BCR 100:1) after preincubation (2 h) of the bacteria with chitosans with different DP and DA. Data represent mean ± SD from *n* = 3 independent experiments with *n* = 2 technical replicates each

Concentration [µg/mL]		Chitosan 134			Chitosan 90/20		
		1000	100	10	1000	100	10
*C. jejuni*-strain	DSM 27585	29 ± 29	37 ± 32	41 ± 30	68 ± 41	64 ± 27	198 ± 218
	Isolate LH 90	37 ± 38	47 ± 46	131 ± 107	84 ± 108	77 ± 58	127 ± 77
	Isolate LH 187	74 ± 50	60 ± 15	111 ± 59	72 ± 23	113 ± 62	118 ± 58

To control this, chitosan 70/20 was tested, which is characterized by high DA (23%) and a DP comparable to 90/20: as expected, no antiadhesive activity was observed. This finding correlates also to the data obtained from chitosan 661 (low DP and DA 20%), which turned out to be completely inactive. Based on these data, high DP/low DA chitosans 114 and 134 were selected for further investigations. Both polymers exert strong antiadhesive potential and especially chitosan 134 reduces bacterial adhesion significantly at the 100 μg/mL level by about 60%. It has to be mentioned that both chitosans, 114 as well as 134, can be considered in principle as more or less high molecular glucosamine polymers with nearly no acetylation.

Chitosans 134 and 90/20 were further investigated in more detail against the *C. jejuni* DSM 27585 and *C. jejuni* field isolates LH 90 and LH 187. Again quite standard deviations were disturbing, due to agglomeration of the bacteria by the chitosans, and this also disabled testing on statistical significance between the different test groups. Again, the tendencies were clear: Both chitosans exerted antiadhesive activity at ≥ 100 μg/mL. Interestingly, the 90/20 derivative had a tendency to agglomerate the bacteria to the host cells at the low concentration of 10 μg/mL, a phenomenon which was also observed for chitosan 134, but to a lesser extent.

Based on these findings, chitosan 134 was selected for the subsequent investigations.

To investigate if the monomeric building blocks of chitosan, glucosamine and N-acetylglucosamine, contribute to the antiadhesive effect, the influence of both monomeric carbohydrates was tested at 1 mg/mL, but no inhibition of bacterial adhesion was found (data not shown), suggesting that high molecular weight is a prerequisite for antiadhesive activity.

To ensure that the observed antiadhesive effects of chitosan 134 is due to an interaction with *C. jejuni* and not to changes of the host cells, Caco-2 cells were pretreated for 2 h (100 and 1000 μg/mL). After removal of excess polysaccharides by washing and incubation of the host cells with *C. jejuni* DSM 27585, the relative bacterial adhesion was determined. No significant changes in *C. jejuni* host cell interaction were observed (data not shown). From these experiments it is deduced that chitosan 134 interacts with *C. jejuni* and not with the eukaryotic host cells in regard to the adhesion.

**Fig. 1:**
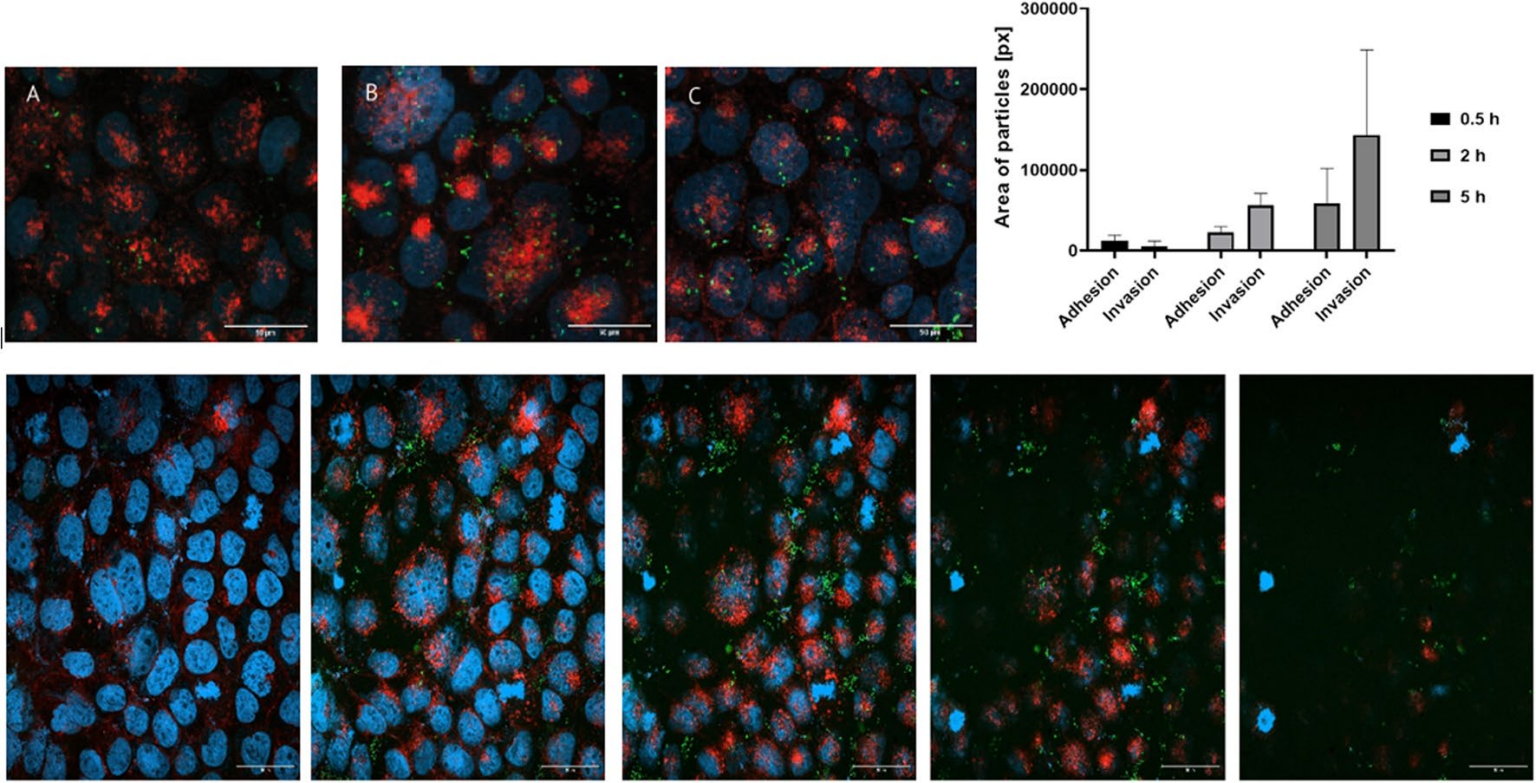
Confocal laser scanning microscopy of Caco-2 cells, incubated for 0.5 (**A**), 2 (**B**) and 5 h (**C**) with *C. jejuni* (BCR 100:1). Quantitative evaluation by pixel counting of green fluorescence CFDA-SE stained bacteria over the time clearly shows strong invasion after 5 h (**D**). Different z-stack sections reveal representative depth sections of infected Caco-2 cells after 5 h of incubation (**E** to **I**) differentiating between adhering and internalized *C. jejuni*. *C. jejuni* cells are displayed in green after staining with CFDA-SE, cell nuclei of T24 cells (blue) are stained with DAPI, and wheat germ agglutinin stain host cell membranes with AlexaFluor 594 (red)

From these investigations, antiadhesive effects for the low DA/high DP chitosans were obvious, but due to the problems with clustering of chitosan-treated bacteria and the resulting high standard deviations, a second, independent assay protocol was to be established to confirm the antiadhesive effects. Therefore, adhesion imaging by confocal laser scanning microscopy (CLSM) was used. Caco-2-cells were cultivated on collagen-coated slides to a confluence of 80 to 90%. Triple staining was performed with DAPI for visualization of the cell nuclei, cell membranes were stained with WGA Alexa-Fluor594 conjugate, and *C. jejuni* was stained by CFDA-SE. Interaction of *C. jejuni* with the Caco-2 cells at BCR 100:1 was investigated after different times of incubation (0.5, 2, and 5 h) (Fig. 1A to C). Location of FITC-labelled bacteria can clearly be assigned to occur on the outer side of the host cells, but during a prolonged incubation time of 5 h also internalization gets obvious (Fig. 1D). For quantitative evaluation *z*-staging was performed in different depth levels, and the relative amount of labelled bacteria was determined. As displayed in Fig. 1E to I, a time-dependent adhesion and invasion process was observed: while after 0.5 h bacteria which were adhering to the cell membrane were dominant, the amount of internalized bacteria increased strongly after 2 and 5 h. No relevant differences in the amount of adhering bacteria between 2 and 5 h were observed. Pretreatment of *C. jejuni* (2 h) with different chitosans, followed by incubation of the labelled and chitosan-treated bacteria with Caco-2 cells at a BCR 1001 over 1 and 2 h, dramatically decreased the relative amount of adhering bacteria (Table 5). As expected from the previous experiments chitosan 134 had strongest inhibitory effect, while the antiadhesion was less for chitosan 114, and also chitosan 80/20 exerted only moderate effect. Also the amount of internalized bacteria was strongly reduced by the chitosans. From these data, the antiadhesive effects of chitosans, which had been observed by the flow cytometric assays, have been clearly confirmed by CLSM. Comparison of the relative amounts of adhering and invaded bacteria to and into the host cells indicates that the reduced bacterial internalization after chitosan treatment is probably not due to a specific anti-invasive mechanism, but is based on the reduced bacterial adhesion to the host cells. To pinpoint potential *Campylobacter* surface structures which are influenced by the antiadhesive chitosans, selected bacterial adhesins were investigated. Main outer membrane protein adhesins of *C. jejuni* with binding capacity to epithelial cells are CadF, FlpA, JlpA, and CapA (Kemper and Hensel 2023; Kreling et al. 2020). The two adhesins FlpA (fibronectin-like protein A) and JlpA (*Jejuni* lipoprotein A) were selected in order to investigate the influence of chitosan on the interaction of the adhesins with the corresponding binding partners of the host cell using recombinant proteins and a specific immunoassay. FlpA is known to interact specifically with the peptide sequence (WRPHPDFRV) of the second FNII 45 kD gelatine binding domain of fibronectin (FN) (Flanagan et al. 2009; Larson et al. 2013). This binding leads to the release of the Cia effector protein (*Campylobacter* invasion antigen), integrin activation, Erk1/2 activation of the host cell, and subsequent invasion of *C. jejuni* into the host cell (Talukdar et al. 2020; Konkel et al. 2020). JlpA is a 42 kD glycosylated lipoprotein that binds to heat shock protein HSP90α, which can be found within the extracellular matrix and on cell surface of the host cells, which again activates NF-κB-dependent inflammatory response (Jin et al. 2001, 2003; Kawai et al. 2012).

**Table 5:** Influence of preincubation (2 h) of *C*. *jejuni* with different chitosans on the relative adhesion to and invasion of Caco-2 cells (BR: 100:1) after 1 and 5 h of incubation time as determined by confocal laser scanning microscopy and quantification of CFDA-SE labelled bacteria by evaluation of the respective pixel area. *UC*, untreated control after 1 h of incubation time (= 100% using the adherent bacteria). Data are related to evaluation of a single experiment

	Conc. [µg/mL]	1-h incubation		5-h incubation	
		Rel. adhesion [%]	Rel. invasion [%]	Rel. adhesion [%]	Rel. invasion [%]
UC		100	56	85	134
Chitosan 134	1000	11	3	12	7
	100	0	0	4	3
Chitosan 114	1000	8	4	2	6
	100	22	6	16	14
Chitosan 80/20	1000	37	9	8	6
	100	48	3	17	6

For recombinant expression, both adhesins *flpA* and *jlpA* from *C. jejuni* DSM 27585 were amplified by PCR and fused cloned into a vector adding a 6 × His tag. Sequence alignment of the 1142 bp (*flpA*) and 1089 bp (*jlpA*) products to the reference sequences confirmed identity (Supplementary Figures S1 and S2). The coding sequences were cloned into the cold shock expression vector *pCOLD I*, and the proteins were expressed under cold shock in *Escherichia coli* ArcticExpress. Purification was performed by immobilized metal chelate affinity chromatography (IMAC). The amino acid sequence was investigated by mass spectrometry for the tryptically digested peptides. For FlpA, 94% homology and for JlpA 100% homology was determined. Thus, the identity of the so obtained proteins with the expected amino acid sequence of FlpA (UniProt accession no.: Q0P8X7) and JlpA (UniProt accession no.: P45492) from *C. jejuni* DSM 27585 is given (Parkhill et al. 2000; Parrish et al. 2007).

The process was upscaled to 1 L batches. *E. coli* ArcticExpress with *flpA* or *jlpA* in *pCOLD* plasmid was cultured in 5 mL LB medium containing 10 µg/mL ampicillin O/N at 37 °C. Cultures were transferred to 1 L of LB medium without antibiotic supplementation and incubated for 3 h at 30 °C to an OD_600_ of 1.2. The temperature was adjusted to 11.5 °C and 1 mM IPTG added. Cultures were incubated for 48 h at 11.5 °C for protein expression. Successful expression was verified by SDS-PAGE, followed by Western blot with anti His-tag immunostaining. Again, target proteins were purified from the bacterial lysate by IMAC. FlpA and JlpA containing fractions were pooled and dialysed to yield 2.42 mg/mL FlpA and 2.56 mg/mL JlpA.

**Fig. 2:**
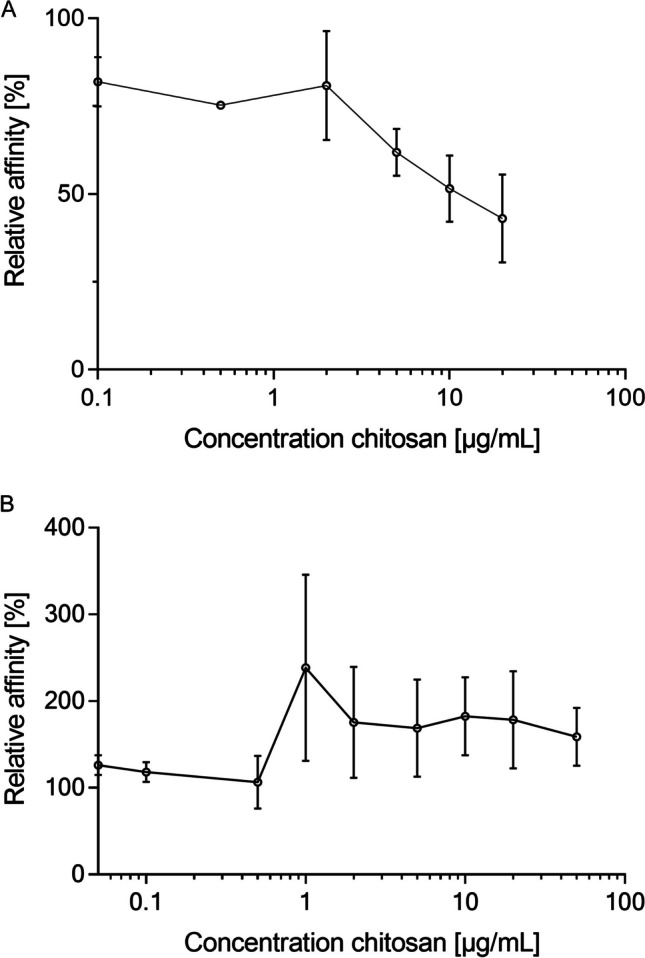
Relative binding affinity of **A** recombinant JlpA (0.5 µg/well) to Hsp90α and **B** recombinant FlpA (7.5 μg/well) to FN under coincubation with chitosan 134. Data represent the mean ± SD from *n* = 3 independent experiments with *n* = 2 technical replicates

Subsequently, a specific in-house sandwich ELISA was established to investigate a potential interaction of chitosan 134 against the binding of *C. jejuni* adhesins FlpA and JlpA to the ligands FN and Hsp90α. 96 well plates were coated either with FN or Hsp90α. His-tagged FlpA or JlpA specifically bind to their immobilized ligands and can be detected by the anti-His-antibody, which again is detected by a horse-radish-peroxidase (HRP) coupled antibody. HRP/H_2_O_2_ catalyses oxidation of tetramethylbenzidin to blue coloured charge-transfer complexes, which are quantified at *λ* = 450 nm. The obtained absorption values correlate to the amount of FlpA or JlpA which is bound to the immobilized ligands. The so obtained absorbance values were expressed as the relative binding affinity of FlpA and JlpA to the complementary binding partners. For validation of the assay, anti-FN and anti-Hsp90α antibodies were used, which blocked the binding of the adhesins. Also control groups using bovine serum albumin instead of FN or Hsp90α proved absence of unspecific antibody binding. Investigation of potential influence of chitosan 134 revealed no effects against FlpA, but strong and concentration-dependent inhibition of the binding activity of JlpA to Hsp90α (Fig. 2). The EC_50_ of the polymer against JlpA was determined with 2.9 μg/mL.

From these data, it is obvious that low-DA/high-DP chitosan exert antiadhesive activity of *C. jejuni*, which is mediated by an interaction with at least one major adhesin of the bacterium, namely JlpA. The question arose, if this finding might have a practical impact for food processing. Therefore, it was investigated if treatment of commercial chicken meat with chitosan 134 may have an influence on the *Campylobacter* load.

Within a first experiment, three different chicken meat samples (samples #1, #2, and #3) were obtained from different sources (slaughterhouse, farm shops). All three samples, representing biological replicates, were analysed on the occurrence of *Campylobacter* by culturing cultivation on selective Columbia-Blood Agar, followed by multiplex PCR (*16sRNA* as internal control, *hipO* and *asp*). *Campylobacter* was not detected in sample #1, but contamination with *C. jejuni* and *C. coli* was detected in #2 and #3 (CFU counting of sample dilutions see Table 6).

**Table 6:** Influence of a 2-h incubation of chicken meat with chitosan 134 on the *C. jejuni* load. Quantitative evaluation was performed by CFU counting. Data were generated from 3 independent experiments with meat samples #1, #2, and #3 from different commercial origins. Group designations: (− / −) samples without any treatment; (–/ +): preincubation of samples with chitosan 134 (2 ml of a 10 mg/mL solution); (+ / −): preincubation of samples with *C. jejuni*; (+ / +): preincubation of samples with *C. jejuni*, followed by incubation with chitosan 134. *n.a., not evaluable, as CFU > 1000; *n.s.*, not significant. Preincubation with *C. jejuni* DSM 27585 (+ /–and + / +) was carried out with 1 mL of a cell suspension of an OD_600_ = 0.2, corresponding to approx. 1.2 ⨯ 10^8^ CFU. Significance was investigated by unpaired *t*-test

Incubation with:		Sample (*n*)	Dilution		
*C. jejuni* DSM 27585	Chitosan 134		1:10	1:100	1:1000
–	–	1	0	0	0
		2	n.a.*	89	3
		3	n.a.*	5	1
Mean ± SD			666 ± 577	31 ± 50	1.3 ± 0,8
–	+	1	0	0	0
		2	0	0	0
		3	0	0	0
Mean ± abs. SD			0	0	0
Significance (− / +) *vs.* (− / −)			n.s. *p* = 0.1161	n.s. *p* = 0.3388	n.s. *p* = 0.2051
+	–	1	n.a.*	161	
		2		ca. 500	
		3		n.a.*	
Mean ± SD			1000	1000	554 ± 422
+	+	1	243	19	7
		2	0	0	0
		3	ca. 400	149	53
Mean ± SD			214 ± 201	56 ± 81	20 ± 29
Significance (− / +) *vs.* (− / −)			*p* = 0.025	*p* < 0.0001	*p* = 0.0942

In parallel a second treatment group (designation − / +) was prepared, for which sample #1, #2, and #3 were treated with chitosan 134 (2 mL of a 10 mg/mL solution per gram of meat sample), which was spread over the meat pieces, followed by 2-h incubation.

In parallel, a third treatment group (designation + / −) was prepared, for which sample #1, #2, and #3 were inoculated with *C. jejuni* DSM 27585 (1.25 × 10^8^ CFU per gram of meat) and incubated for 1 h.

In parallel, a fourth treatment group (designation + / +) was prepared, for which sample #1, #2, and #3 were inoculated with *C. jejuni* DSM 27585 and incubated for 1 h. Subsequently, chitosan 134 was spread over the meat pieces, followed by 2-h incubation.

The results of CFU counting are displayed in Table 6. Interestingly, in none of the three samples #1, #2, and #3 within the chitosan 134 treated (− / +) group *Campylobacter* was detected. As expected the *Campylobacter* load in the *C. jejuni* inoculated group (+ / −) was extremely high, and exact CFU counting was only possible for the highest dilution. Significant reduction of *Campylobacter* load was monitored in the *C. jejuni*/chitosan-treated group (+ / +). From these data, it is obvious that 2-h treatment of chicken meat with chitosan 134 significantly reduces the bacterial load. It is obvious that the antiadhesive compounds can have positive influence on the *Campylobacter* load of contaminated meat, and this might have practical consequences for meat production or meat processing technology.

**Fig. 3:**
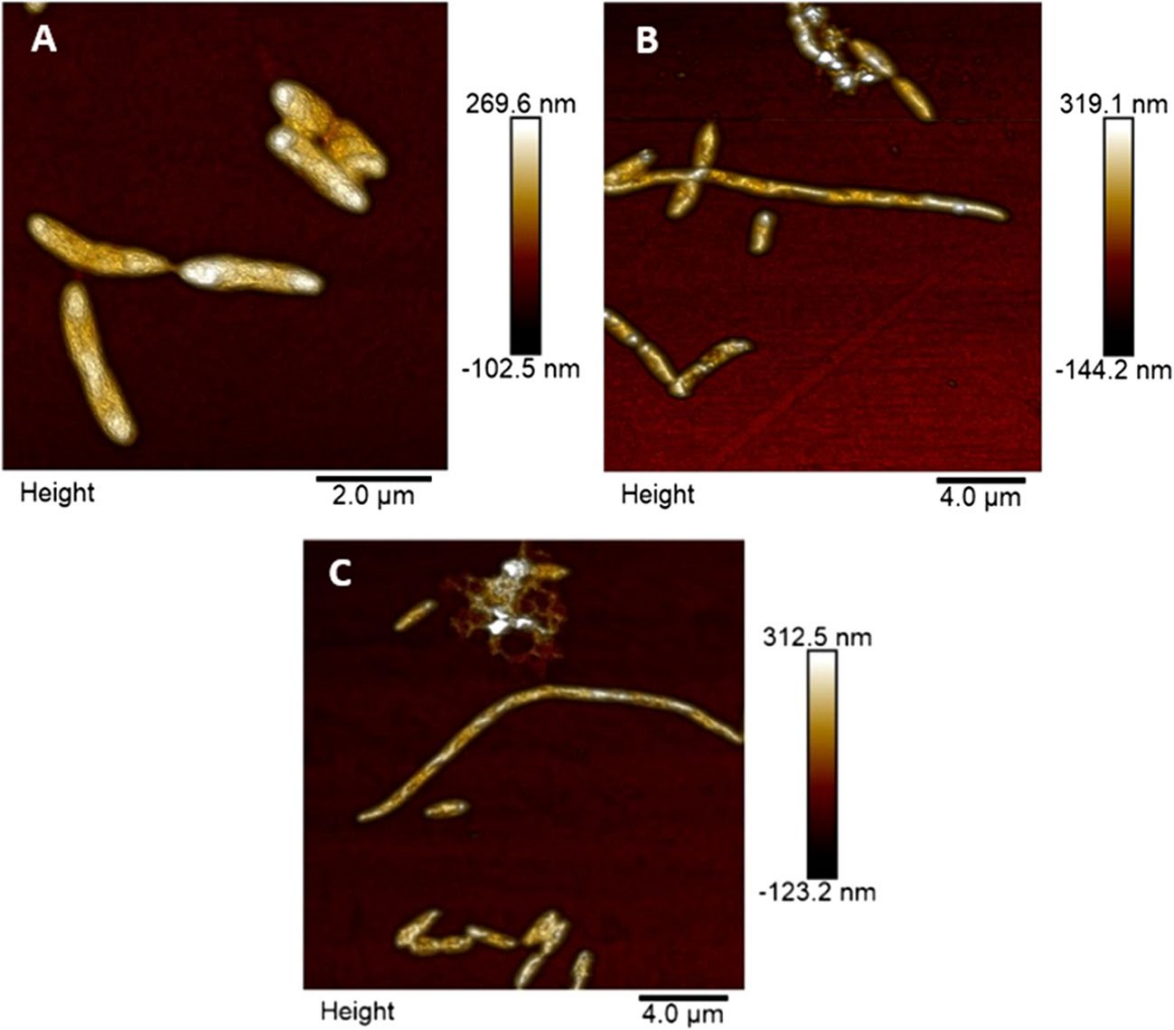
Atomic force microscopy of *C. jejuni* cells under ambient conditions in air. Representative ultrastructural morphology of untreated *C. jejuni* cells (**A**) compared to chitosan 114 (**B**) and chitosan 134 (**C**) treated bacteria at 100 µg/mL. Elongated cells were frequently observed in chitosan-treated individuals at 100 µg/mL, indicating potential cell wall defects induced by chitosan treatment

**Fig. 4:**
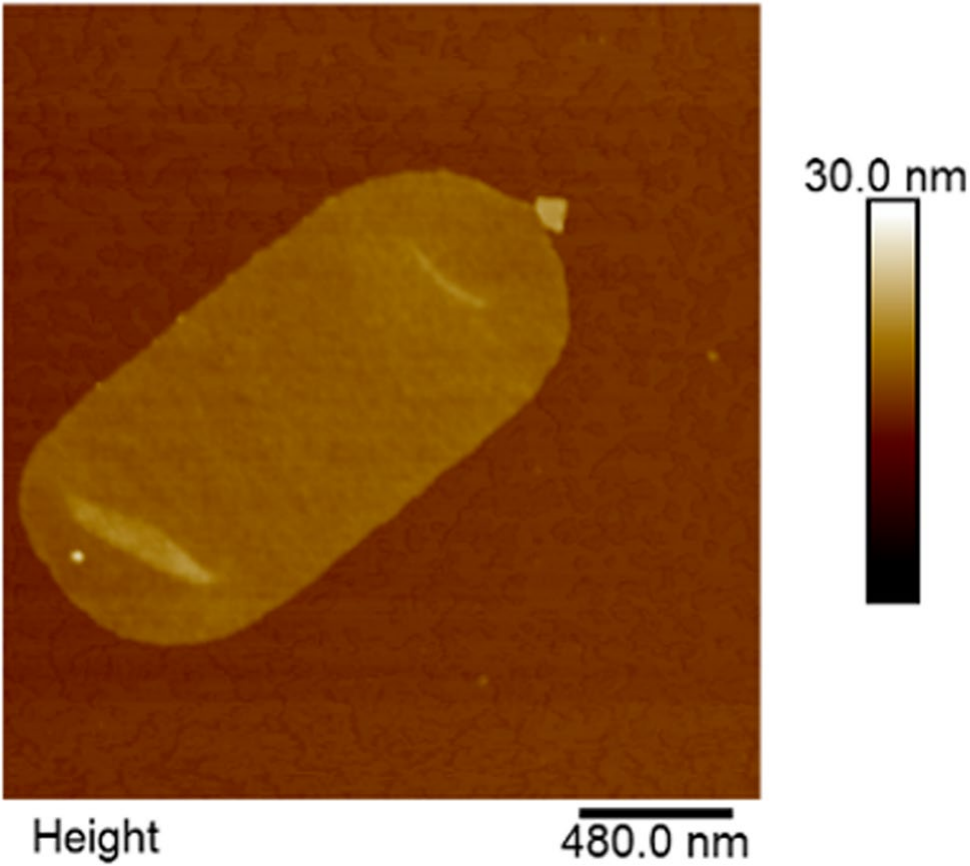
Atomic force microscopy of isolated *C. jejuni* peptidoglycan sacculi under ambient conditions in air. Representative ultrastructural morphology of an isolated peptidoglycan sacculus from an untreated *C. jejuni* cell. In case of chitosan treatment, peptidoglycan isolation procedures yielded agglomerated specimens and did not allow closer insights into peptidoglycan ultrastructure by AFM. Hence, no representation of a chitosan-treated *C. jejuni* sacculus is shown

At this point of the investigations, a further aspect was raised. As the antiadhesive chitosan, 134 might be considered to be a more or less pure β-D-glucosamine polymer, which has nearly no *N*-acetylation; it might be discussed that the positive charge of the polymer is the reason for electrostatic interaction with the negatively cell charged surface, especially with the protein-rich outer membrane of *C. jejuni*. As acetylation of chitosans (which correlates with reduced positive charge) will decrease the antiadhesive potential, it was interesting to investigate if morphological changes of *C. jejuni* can be monitored after treatment with chitosans. According to the literature, interaction of chitosan to phospholipids is discussed (Liu et al. 2004). Also, potential damages of the barrier function of the outer membrane by chitosans have been reported, which might explain the observed antiadhesive effect via JlpA (Helander et al. 2001). To investigate a potential influence of the antiadhesive chitosans on the morphology of *C. jejuni*, the bacteria were treated with the polymers 134 and 114 (1000 and 100 μg/mL), followed by atomic force microscopy (AFM) of immobilized cells under ambient conditions in air. Interestingly, strong elongation of the bacteria up to the three- to fourfold length) was observed in the 100 μg/mL group (Fig. 3). Quantification of the amount of elongated cells was not possible in details due to the strong formation of aggregates, but typically, the formation of non-divided cells was observed for the chitosan-treated groups (a typical chitosan-induced bacteria cluster with outgrowing elongated bacteria is displayed in Supplementary Figure S3). Typically, similar types of elongation have been described for bacteria with damaged cell wall formation (e.g. by fosfomycin or β-lactam antibiotics (Elsbroek et al. 2023). This finding indicates that low acetylated chitosans interact with the correct cell wall formation, which again could explain also changes in the outer membrane. As cell wall architecture can be monitored more specifically by isolation and preparation of the murein sacculi of the bacteria and subsequent AFM imaging (Elsbroek et al. 2023), those were isolated and purified from untreated *C. jejuni* (Fig. 4). AFM inspections gave distinct insights into the murein architecture. Unfortunately, this was not possible with chitosan 134 and 114 treated *C. jejuni*. Despite manifold experiments, purification of the sacculi was not successful, as a certain part of the polysaccharides sticks to the sacculi preparation, and it was not possible to remove this slimy and viscous layer from the agglomerated material in order to perform unambiguous AFM investigations on single isolated peptidoglycan sacculi. From these results, it is deduced that cell wall formation is disturbed by the low acetylated chitosan, leading to damages in cell division.

## Discussion

As infections with different species from the *Campylobacter* genus are one of four global causes of severe diarrhoeal diseases with a high mortality, the search for antibacterial strategies against these pathogens has to be intensified. Beside, classical antibiotic treatment alternative strategies focus on interaction with typical virulence factors of *Campylobacter*. Especially targeting the initial steps of host–pathogen interplay by inhibition of adhesion to and invasion into the host cells might provide valuable tools against *Campylobacter*, especially in respect to the high infection rates of animals with the pathogens in food production. The use of feed additives with antiadhesive activity for, e.g. for poultry farming, could contribute to reduced infection rates of the animals (Fernandez et al. 2000; Lengsfeld et al. 2007). Chitosan might be a suitable polymer to be considered in poultry farming due to biodegradability, antimicrobial, nontoxicity, and low costs of the raw material (Elnesr et al. 2022). On the other side, chitosans are a quite divers group of glucosamine polymers differing in molecular weight and degree of acetylation but also in the respective pattern of acetylation which characterizes the distinct fine structure of the polysaccharides. The present investigation clearly indicated inhibitory effects of high DP/low DA chitosans against the adhesion of *C. jejuni* to Caco-2 cells. Polymers with higher DA seem to have less effect on the bacterial entry into the cell, but have slight antiproliferative effects. This corresponds to reports that high molecular and low acetylated chitosan, partly positive charged chitosan, interact with the negatively charged bacterial cell walls, covering the prokaryotic surface and inhibiting the interaction with the host cells (Raafat et al. 2008). On the other side, confocal microscopic and AFM studies did not give evidence that the bacterial surface is really covered with a mucilage layer, but relevant changes in the morphology of the bacteria got obvious after chitosan treatment by AFM investigations of the *Campylobacter* sacculi. These investigations suggest that chitosans could interact with *C*. *jejuni* via the outer membrane or the peptidoglycan sacculus, which could explain the lysis of the bacteria after treatment with chitosans. Through this interaction, the equilibrium of the cell wall dynamics could be disturbed which might lead to the immobilization or extraction of peptidoglycan components by chitosans, which has implications for the diffusion of proteins as well as on molecular mechanisms within the cell membrane (Raafat et al. 2008). Interestingly, CLSM of chitosan-treated bacterial indicated that the antiadhesive effects can be correlated also to reduced invasion into the host cell, but quantitative evaluation proves that this is solely to the reduced bacterial adhesion. From this, chitosan cannot be assessed as a specific anti-invasive polymer, only as an antiadhesive polysaccharide. This seems interesting as for some other saccharides, strong anti-invasive effects against *C. jejuni* have been described (Lane et al. 2012). Interestingly, the antiadhesive chitosans seem to interact with the constitutively expressed *Campylobacter* surface adhesin JlpA, inhibiting the binding to its endogenous ligand HSP90α. As no effect of the polysaccharide to the surface adhesion FlpA was observed, this could indicate a certain specificity of chitosan against selected outer membrane proteins of *Campylobacter*. HSP90α exists as surface-exposed or as cytosolic protein (Jin et al. 2003; Pratt 1997), which — among other effects — activates matrix metalloproteinase 2, which again changes extracellular matrix, opens tight junctions, and therefore leads to translocation of *Campylobacter* into the host cells (Eustace and Jay 2004). Due to the interaction of recombinant JlpA with chitosan, it is assumed that this outer membrane protein has lectin-like properties, which might pave the way to identify other carbohydrates with affinity to this bacterial adhesin. As it is known that immunization of chicken against JlpA substantially protects the host against enteric colonization of *C. jejuni* (Gorain et al. 2020), the inhibition of this protein by JlpA blocker administered by food additives to the chicken could provide a way to reduce *Campylobacter* load in the animals.

In conclusion, our data indicate promising antiadhesive and anti-invasive potential of high molecular weight, strongly de-acetylated chitosans for reducing *C*. *jejuni* load in livestock and food production.

## Supplementary Information

Below is the link to the electronic supplementary material.

Supplementary file1 (PDF 6328 KB)

## Data Availability

The authors declare that the data supporting the findings of this study are available within the paper. Should any raw data files be needed they are available from the corresponding author upon reasonable request.
