# Supplementary material for: High molecular/low acetylated chitosans reduce adhesion of Campylobacter jejuni to host cells by blocking JlpA
Source: Appl Microbiol Biotechnol. 2024 Jan 24;108:171. doi: 10.1007/s00253-024-13000-0 (PMC10810038; doi:10.1007/s00253-024-13000-0)
Supplement: Supplementary file 1 [file 253_2024_13000_MOESM1_ESM.pdf]

SUPPLEMENTARY DATA

High molecular / low acetylated chitosans reduce adhesion of *Campylobacter jejuni* to host cells by blocking JlpA

Vanessa Kreling[1], Franco H. Falcone[2], Fabian Herrmann[1], Leon Kemper[1], Daniel Amiteye[1], Stefan Cord-Landwehr[3], Corinna Kehrenberg[4], Bruno M. Moerschbacher[3] and Andreas Hensel[1*]

[1] University of Münster, Institute of Pharmaceutical Biology and Phytochemistry, Corrensstraße 48, 48149 Münster, Germany.
ORCID: 0000-0003-0886-8384

[2] Institute of Parasitology, Justus Liebig University Giessen, Schubertstraße 81, 35392 Giessen, Germany

[3] University of Münster, Institute of Plant Biology and Biotechnology, Schlossplatz 8, 48143 Münster, Germany. ORCID 0000-0003-1915-1936 (SCL), 0000-0001-6067-3205 (BMM)

[4] Institute of Veterinary Food Science, Justus Liebig University Giessen, Frankfurter Straße 92, 35392 Giessen, Germany

*** Correspondence:**
Andreas Hensel, Institute of Pharmaceutical Biology and Phytochemistry, University of Münster, Corrensstraße 48, 48149 Münster, Germany

Phone: +49 251 8333380, e-mail: ahensel@uni-muenster.de

Table S1: Primers for amplification of the *C. jejuni* genes *flpA* and *jlpA*, for the cold shock expression plasmid pCOLD I, and for multiplex PCR for identification of *Campylobacter*.

Table 2: Primers for amplification of the *C. jejuni* genes *flpA* and *jlpA*, for the cold shock expression plasmid pCOLD I, and for multiplex PCR for identification of *Campylobacter*.

	**Sequence (5´- 3´)**
Amplicon: *flpA* in pCOLD I	
flpA_NdeI-FOR	TATATCATATGAGCTTGCCAAAGGTTGAAAG
flpA_XbaI-Rev	ATATATCTAGAGACTGAGCCGCCTTAACTTTG
Amplicon: pCOLD I (sequencing primers)	
pCOLDI FOR	ACGCCATATCGCCGAAAGG
pCOLDI REV	GGCAGGGATCTTAGATTCTG
**Amplicon: *jlpA* in pCOLD I**	
jlpA_NdeI-FOR	CCCGGGCATATGTGCGGAAATTCCATAG
jlpA_PstI-REV	ATATATCTGCAGAAATGACGCTCCGCCCA
16S rDNA	GGGAGGCAGCAGTRRGGAAT
	TGACGGGCGGTGRGTACAAG
asp Aspartokinase	GGTATGATTTCTACAAGCGAG
	ATAAAAGACTATCGTCGCGTG
hipO Hippuricase	GACTTCGTGCAGATATGGATGCTT
	GCTATAACTATCCGAAGAAGCCATCA

Table S2: Relative proliferation [%] of *C. jejuni* after 48 h ($t_{1/48h}$) under chitosan influence and 48 h ($t_{2/96h}$) after changing to test compound-free, unsupplemented media relative to the untreated control UC ($t_{1/48h}$ value = 100 %). The UC after 96 h ($t_{2/96h}$ value) had a relative proliferation of 181 ± 17 % (related to the ($t_{1/48h}$ value = 100 %). Relative values represent the mean ± SD from n = 3 independent experiments with n = 4 technical replicates.

**Chitosan**		**Concentration [mg/mL]**		
	***t* [h]**	**5**	**2.5**	**1.25**
		Relative proliferation [%] UC $t_{1/48h}$= 100%; UC $t_{2/96h}$ = 181 %		
**134**	$t_{1/48h}$	0 ± 0	54 ± 63	74 ± 40
	$t_{2/96h}$	190 ± 75	176 ± 68	176 ± 10
**114**	$t_{1/48h}$	26 ± 36	10 ± 10	89 ± 55
	$t_{2/96h}$	194 ± 14	100 ± 28	161 ± 48
**652**	$t_{1/48h}$	24 ± 18	33 ± 25	87 ± 59
	$t_{2/96h}$	123 ± 24	137 ± 27	161 ± 18
**651**	$t_{1/48h}$	6 ± 5	6 ± 8	177 ± 123
	$t_{2/96h}$	157 ± 54	119 ± 27	172 ± 70
**661**	$t_{1/48h}$	33 ± 25	137 ± 106	156 ± 91
	$t_{2/96h}$	284 ± 242	273 ± 209	175 ± 136
**90/20**	$t_{1/48h}$	10 ± 13	10 ± 15	181 ± 128
	$t_{2/96h}$	174 ± 45	145 ± 40	197 ± 107
**80/20**	$t_{1/48h}$	10 ± 3	7 ± 5	111 ± 76
	$t_{2/96h}$	177 ± 58	129 ± 42	207 ± 112
**70/20**	$t_{1/48h}$	7 ± 6	1 ± 1	123 ± 92
	$t_{2/96h}$	115 ± 20	120 ± 15	141 ± 50

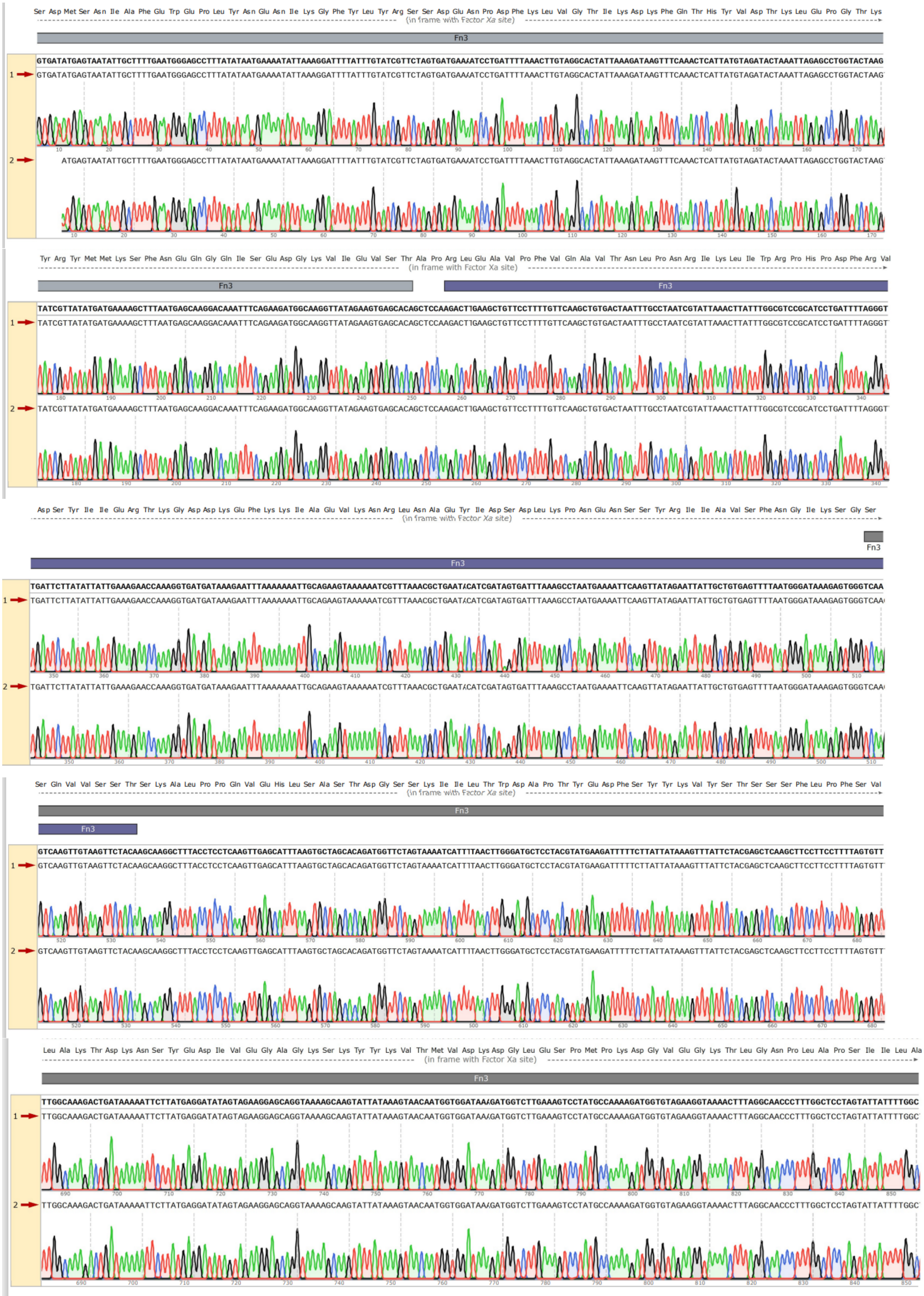

(in frame with Factor Xa site)
Fn3

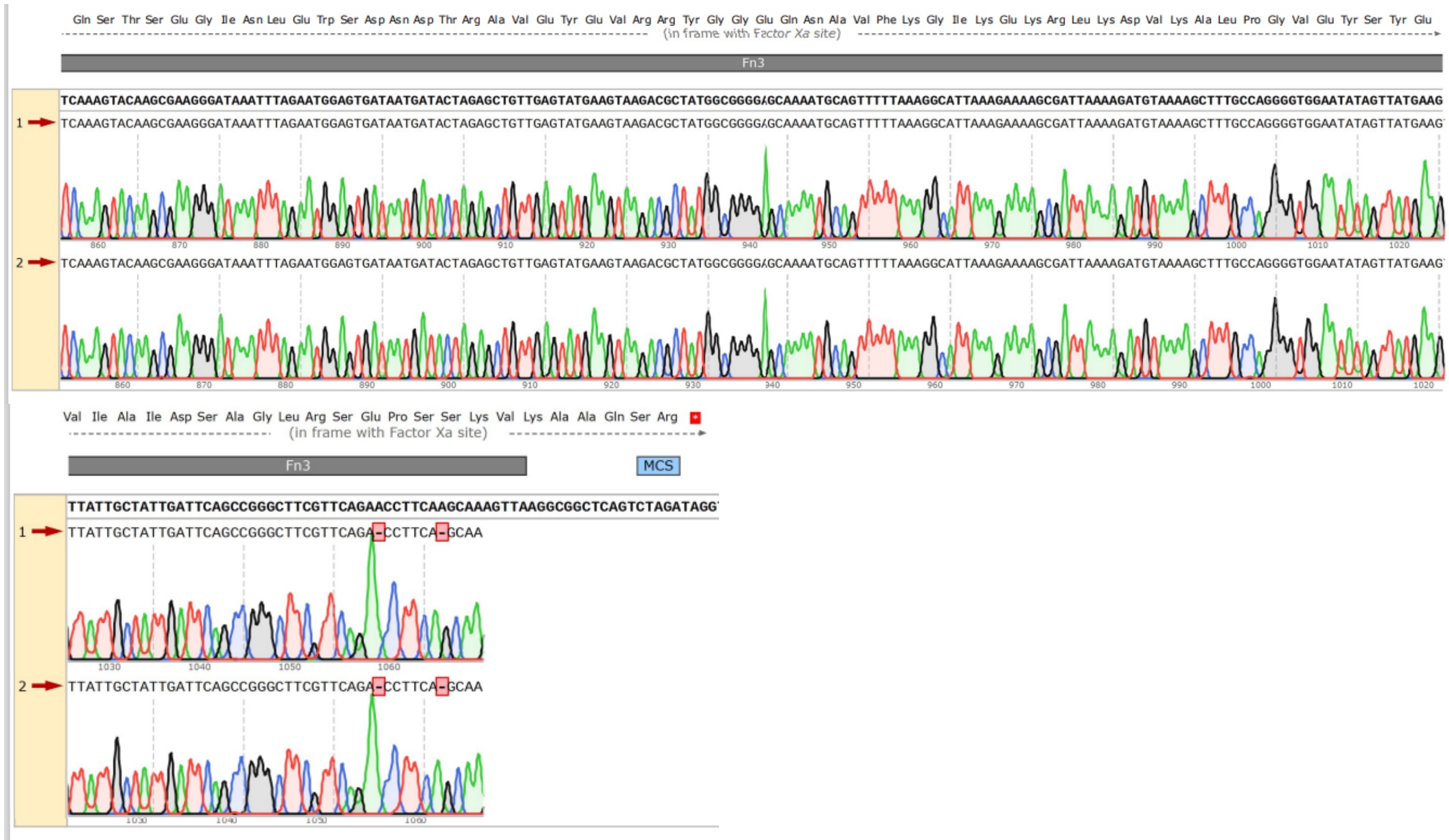

Figure S1: Sequence alignment of the *flpA* gene products of the forward and reverse primers with the theoretical *flpA* sequence for cloning into pCOLD using SnapGene. The reference sequence is shown above in bold letters. (1): Sequence of the PCR product with the forward primer. (2) Sequence of the PCR product with the reverse primer. In the figure, the sequenced bases are indicated by the letter code as well as by color: RED/T: thymine; BLUE/C: cytosine; GREEN/A: Adenine; GREY/G: Guanine. In addition, the coded amino acids are listed in this representation.

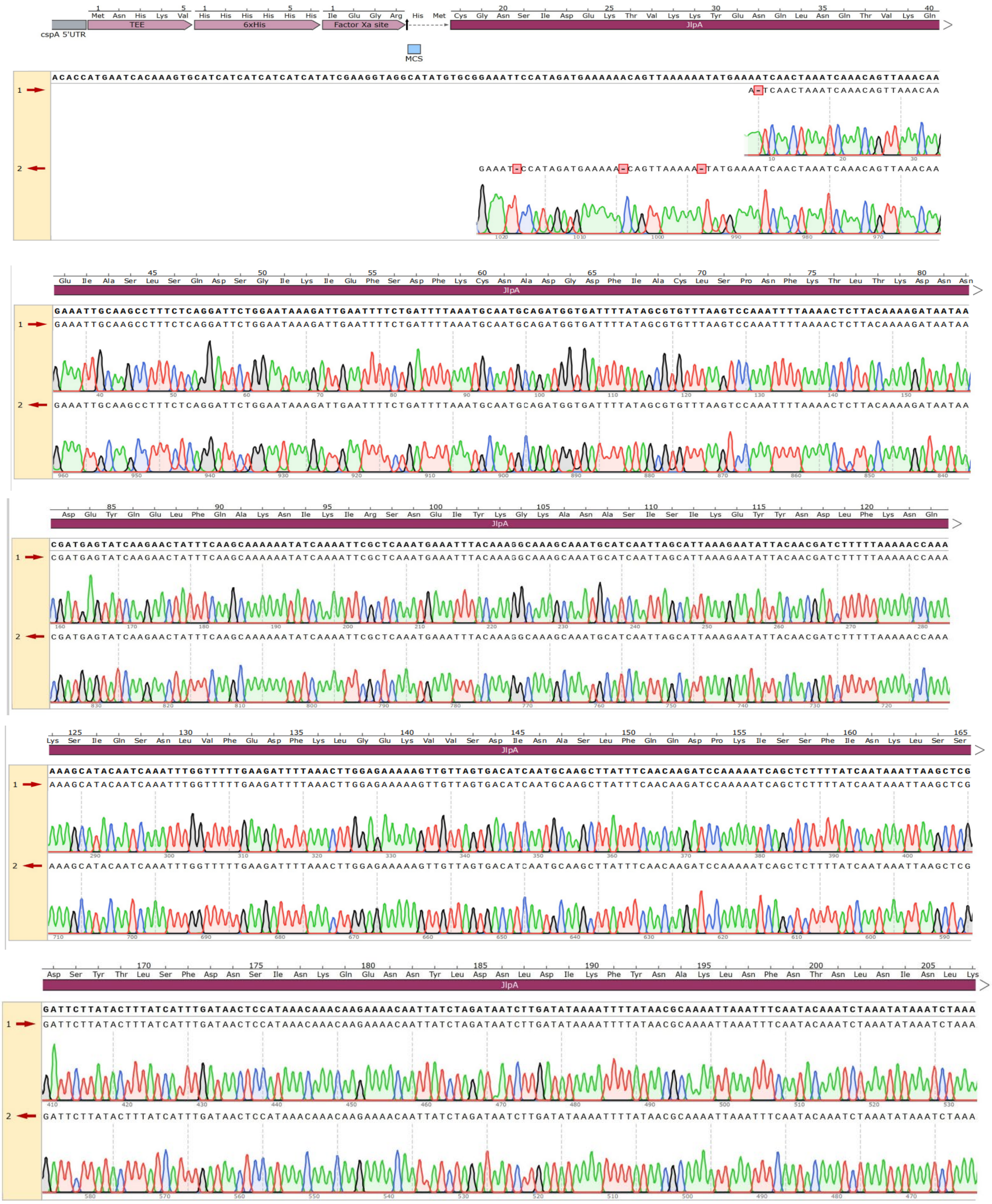
cspA 5'UTR
TEE
6xHis
Factor Xa site
MCS
JlpA

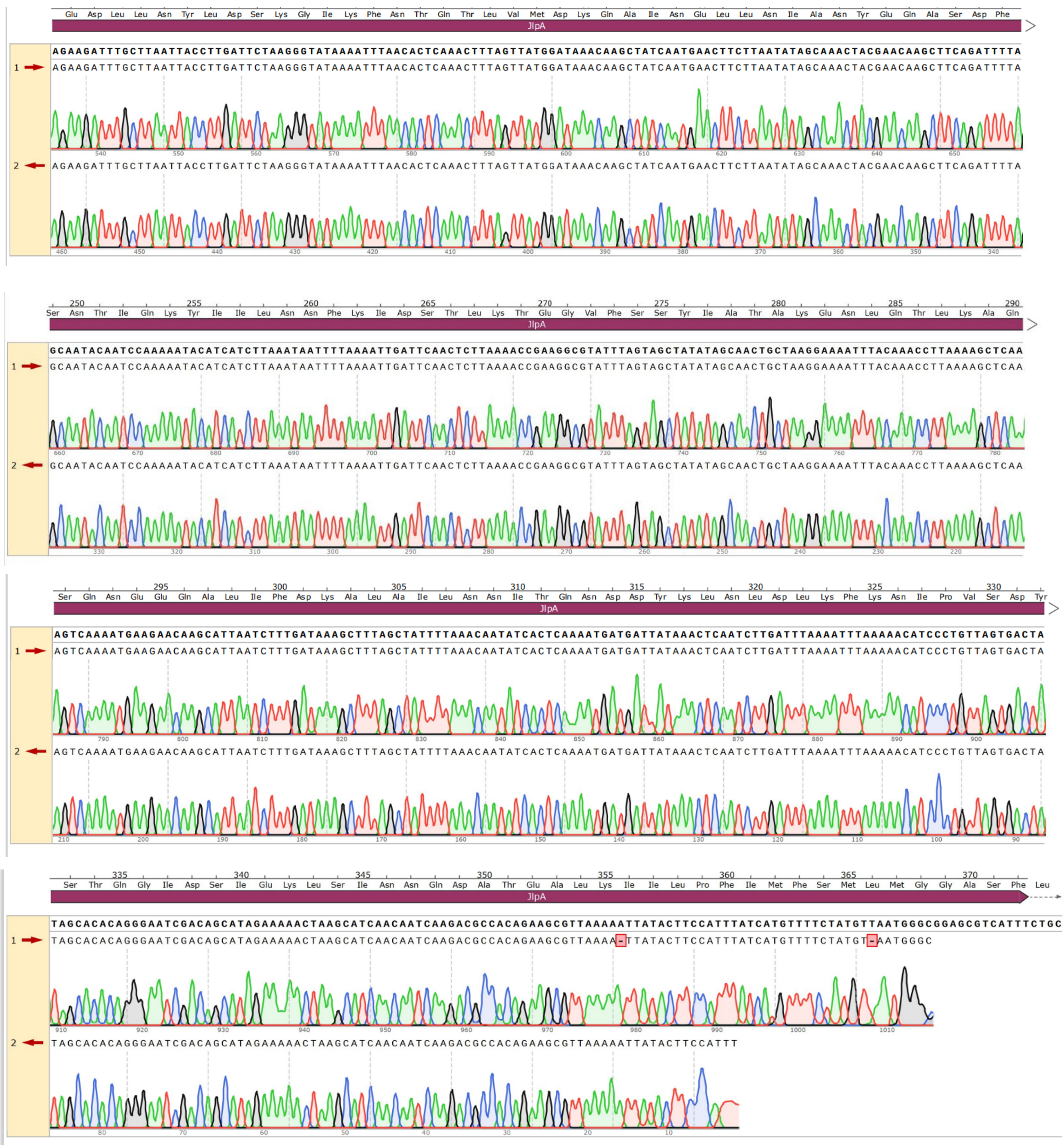

Figure S2: Sequence alignment of the jlpA gene product of the forward and reverse primers with the theoretical jlpA sequence for cloning into pCOLD using SnapGene. Reference sequence is shown above each in thick letters. (1): Sequence of the PCR product with the forward primer. (2) Sequence of the PCR product with the reverse primer. Nucleotides are indicated by the letter code and by color: RED/T: thymine; BLUE/C: cytosine; GREEN/A: adenine; GREY/G: guanine.

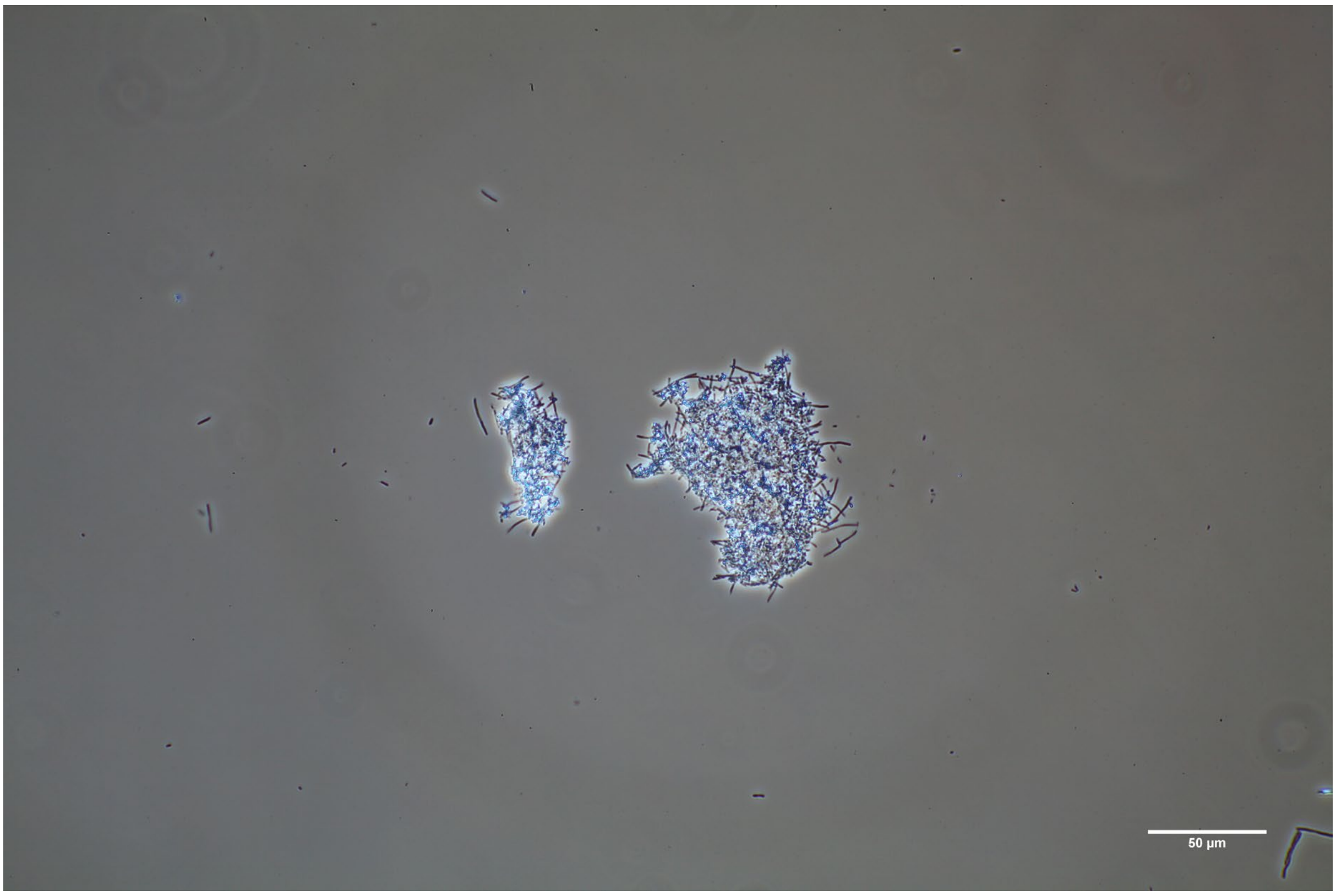

Figure S3: Chitosan 134 treated *Campylobacter jejuni* after 72 h of incubation leads to strong bacterial agglomeration and elongation of bacteria.
